# Recovery of Chlorogenic Acids from Agri-Food Wastes: Updates on Green Extraction Techniques

**DOI:** 10.3390/molecules26154515

**Published:** 2021-07-27

**Authors:** Ilaria Frosi, Irene Montagna, Raffaella Colombo, Chiara Milanese, Adele Papetti

**Affiliations:** 1 Drug Sciences Department, University of Pavia, 27100 Pavia, Italy; ilaria.frosi01@universitadipavia.it (I.F.); irene.montagna01@universitadipavia.it (I.M.); raffaella.colombo@unipv.it (R.C.); 2 C.S.G.I. & Department of Chemistry, Physical Chemistry Section, University of Pavia, 27100 Pavia, Italy; chiara.milanese@unipv.it

**Keywords:** agri-food wastes, waste valorization, sustainability, chlorogenic acids, green extraction techniques, health, nutraceuticals, bioactives

## Abstract

The agri-food sector produces a huge amount of agri-food wastes and by-products, with a consequent great impact on environmental, economic, social, and health aspects. The reuse and recycling of by-products represents a very important issue: for this reason, the development of innovative recovery and extraction methodologies must be mandatory. In this context of a circular economy, the study of green extraction techniques also becomes a priority in substitution of traditional extraction approaches. This review is focused on the recovery of chlorogenic acids from agri-food wastes, as these compounds have an important impact on human health, exhibiting several different and important healthy properties. Novel extraction methodologies, namely microwave and ultrasound-assisted extractions, supercritical fluid extraction, and pressurized-liquid extraction, are discussed here, in comparison with conventional techniques. The great potentialities of these new innovative green and sustainable approaches are pointed out. Further investigations and optimization are mandatory before their application in industrial processes.

## 1. Introduction

Over the last decade, the huge urbanization of the world population has led to an increase in organic waste production, which is no longer sustainable. The Food and Agriculture Organization (FAO) of the United Nations estimated that 1.3 billion metric tons of edible food are lost or wasted every year [1]. The disposal of these by-products causes serious environmental hazards and implies high costs. For these reasons, nowadays, sustaining their impact is a priority. The European Commission developed long-term strategies to reach zero impact by 2050, supporting a circular economy strategy in order to give a second life to waste products [2]. In this context, the valorization of food waste is a fascinating challenge and involves great interest by the scientific community as a possible source of nutraceuticals [3,4,5,6].

**Figure 1:**
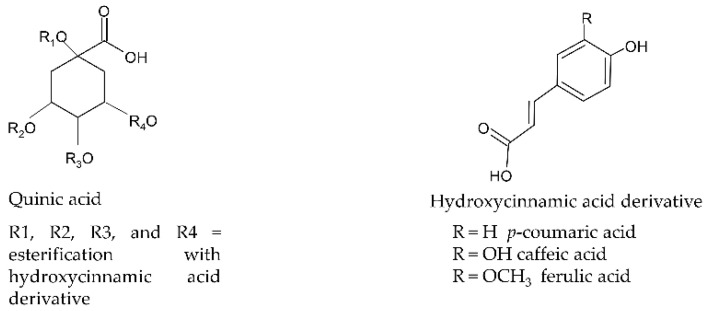
General structures of the main chlorogenic acids (esters between quinic acids and hydroxycinnamic acid derivatives).

Natural products have always been considered attractive thanks to their composition in bioactives. Among them, a prominent role is played by chlorogenic acids (CGAs), a class of phenolic compounds well known for their different biological activities. CGAs are a large family of esters of quinic acid and *trans*-cinnamic acids (mainly caffeic, coumaric, and ferulic acids); up to date, 71 different chemical compounds were identified from different sources [7], and the most representative ones are reported in Figure 1. Caffeoylquinic acids (CQAs), in particular mono- and di-CQAs, are the most widespread in vegetables and plants together with the different isomeric forms of feruloylquinic acids (FQAs). Among CQAs, 5-CQA is the most abundant isomer present in nature, and it has been studied for its widely-known health effects. Convincing results from numerous studies have shown its antioxidant [8], antiviral [9], antibacterial [10,11], and anti-inflammatory [12] properties, resulting in an effective dietary protective phenolic compound. Moreover, clinical studies showed cardio- [13], neuro- [14] and hepato-protective [15] capacities, being a valid ally against chronic and age-related diseases. There is also recent evidence that CGAs may have a role in lipid and glucose metabolism in metabolic disorders, such as dyslipidemia and hyperglycemia. 5-CQA has been shown both to potentially restore the activity of lipoprotein and lipid metabolism regulatory enzymes, improving dyslipidemia, and to modulate insulin and gastrointestinal hormones, attenuating hyperglycemia [15,16,17].

Considering the above-mentioned CGAs health potential, the importance of finding more efficient extraction approaches, using at the same time sustainable, cost-effective, and eco-friendly techniques, is evident. 

The current review provides an update of the most used and promising green extraction techniques for the recovery of CGAs from agri-food wastes, presenting their characteristics vs. conventional approaches. The parameters to be considered for the optimization of the extraction yield are also discussed, focusing on the advantages/disadvantages derived from the use of novel green solvents vs. the traditional ones.

## 2. Chlorogenic Acids in Agri-Food Wastes

It is well known that CGAs are very abundant in plants and foods, including coffee beans, apple, berry fruits, artichoke, potato tubers, and eggplant [15,18]. Due to their beneficial health proprieties, it is useful to find and examine alternative sources of these valuable bioactive compounds and evaluate their potential content in CGAs. Different agri-food wastes have been analyzed, and a great number has been found rich in CGAs and therefore potentially usable as new raw material to obtain these active ingredients. 

In particular, coffee, potato and artichoke waste were found to be good CGAs sources. Coffee is one of the most consumed beverages all over the world; hence, every year, the coffee industry produces tons of by-products, including spent coffee grounds, coffee husk and coffee silverskin [19]. Spent coffee ground is the solid residue remaining after coffee water extraction [20]. Fanali et al. [21] detected fifteen different CGAs in spent coffee grounds extracts, including mono-CQAs (3-CQA, 4-CQA, 5-CQA) and di-CQAs (3,4-diCQA, 3,5-diCQA, 4,5-diCQA), two isomers of caffeoylepiquinic acid, 5-coumaroylCQA, and two isomers of caffeoylferuloylquinic acid. Among them, Yoo et al. [22] reported that 3-CQA is the most abundant, followed by similar amounts of 4-CQA and 5-CQA, while FQAs and di-CQAs were identified in lower quantities. Coffee husk is generated during the dry processing of coffee beans [19]; according to Silva et al. [23], the main identified phenolic compound in this waste is 5-CQA, ranging from 16.64 to 337.07 μg/g. Coffee silverskin is produced during the roasting process [19]; Guglielmetti et al. [24] found out that the total CQAs content in it ranges from 1.06 g/kg dry weight (dw)—using MAE—to 2.68 g/kg by conventional extraction methods. Potato peel is another relevant source of chlorogenic acids and is the major waste derived from the potato processing industry [25]. The study by Riciputi et al. [26] investigating potato by-products from five different potato varieties shows very interesting results: 5-CQA is the most abundant phenolic acid (1.3–4.1 mg/g dw), representing 49–61% of the potatoes’ total phenolic compounds. The total amounts of the other two isomers (4-CQA and 3-CQA) varied from 0.7 to 2.6 mg/g dw, while diCQAs and 5-FQA were less abundant. During the industrial processing of artichoke, about 60–85% of the plant is discarded [27]. Artichoke waste mainly consists of bracts, leaves and stems and has been reported to be rich in CGAs: Punzi et al. [28] identified two different caffeolquinic acids, namely 5-CQA (74.2 ± 4.1 mg/kg dw) and 1,5-CQA (23.2 ± 19 mg/kg dw) in artichoke by-products, including stems, leaves and outer bracts. 5-CQA (3–16 mg/g of extract) and 1,5-diCQA (3–20 mg/g of extract) were also confirmed to be the major phenolic components in artichoke by-products by Pagano et al. [29]. Maietta et al. [30] pointed out the presence of three mono-CQA isomers and two di-CQAs in stem and outer bracts extracts. 1,3-di-CQA and 1,5-di-CQA are predominant in both samples compared to mono-CQA, and in particular, 1,5-di-CQA is the main CGA, detected in concentrations of about 17.4 mg/g dw in outer bracts and 38.7 mg/g dw in stems. Among mono-isomers, 5-CQA is the major extracted phenolic compound (3.7 and 8.9 mg/g dw in outer bracts and stems, respectively) followed by 3-CQA in stem extract and 1-CQA in bract extract. Quite high amounts of CGAs were detected also in blueberry leaves (47.271 ± 0.1803–51.631 ± 0.4703 mg/g dw) [31], almond skin (15.99 ± 0.19 mg/g) [32], carrot pomace (17.79 ± 0.30 mg/g) [33], pomegranate peel (307.26 mg/L) [34], walnut leaves (8.6 ± 0.98–10.7 ± 1.45 mg of 3CQA/g dw) [35], fennel bulbs waste (1.949 ± 0.142 mg of 4CQA/g dw and 0.490 ± 0.035 mg of 3,4-di-CQA/g dw) [36]. CGAs content in tomato skin by-products, depending on the applied extraction method, amounts to 6–62 mg/kg according to Tranfić Bakić et al. [37], 4.05 ± 0.07 mg/kg dw according to Pellicanò et al. [38] and 304.45–454.34 mg/100 g based on the study by Ninčevic Grassino et al. [39].

Additionally, chlorogenic acids were also found in minor quantities in mango peel and leaves, apple by-products, tobacco waste, sunflower by-products, cocoa shell, citrus peel, cauliflower and celery waste.

The content in CGAs of different scraps is summarized in Table 1.

## 3. Conventional Extraction Methods

**Figure 2:**
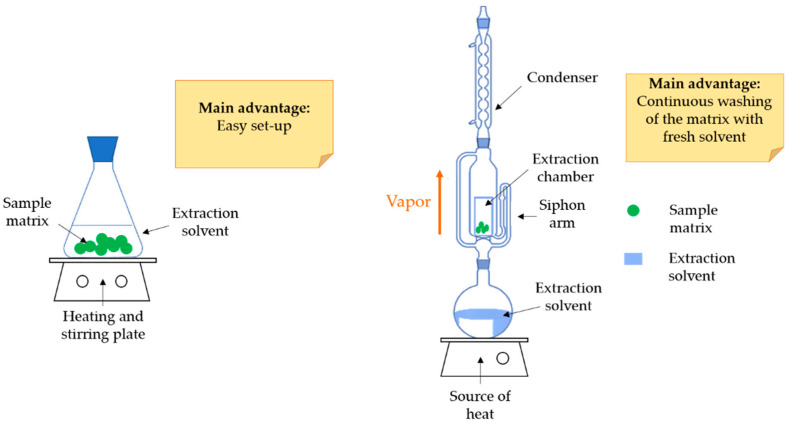
Schematic representation of the main CSE used: maceration extraction (ME) with a source of heat (**left**) and Soxhlet extraction (**right**).

Conventional solvent extraction (CSE) is widely employed for CGAs due to its large applicability, efficiency, and ease to perform. Traditional solid–liquid extraction, such as maceration (ME) and Soxhlet, are the most used techniques for recovering CGAs from food wastes (Figure 2). Maceration is generally performed in a common water bath with the possibility of enhancing the extraction yield by shaking and/or heating. Conversely, Soxhlet extraction requires a specific apparatus, and the main advantage consists in the possibility of repeating the washing step of the waste matrix with fresh solvent aliquots. Basically, both the approaches consist in the exposition of the raw material to different solvents for prefixed times followed by a subsequent filtration of the extract. The extraction yield is strictly correlated to the solvent capacity to penetrate the plant cellular matrix, to solubilize the bioactives, to diffuse into the cellular surface and to transfer the molecules externally to the bulk solution [4,6,58]. 

In solid–liquid extraction, the main variables affecting CGAs recovery are the kind of solvent, the extraction temperature and time, and the solid–liquid ratio. 

Among them, the choice of the solvent is the most relevant key point. CGAs are polar compounds and their solubilization is enhanced by polar solvents, better interacting with their polar sites [23]. Commonly, aqueous-organic (methanol, acetone or ethanol) mixtures are used [52]; ethanol is preferred for nutraceutical purposes because it is generally recognized as safe (GRAS) and therefore suitable for food applications. Hydro-ethanolic mixtures are more effective than pure solvent because ethanol enhances the solubility of the solute, and water facilitates desorption from the sample matrix. Generally, high percentages of ethanol are required to maximize the extraction of CGAs, as reported by Safdar et al. [52], who investigated the effect of different organic solvents (ethanol, methanol, acetone, and ethyl acetate)/water ratio (50/50, 80/20, and 100/0, *v/v*) on the recovery of CGAs from citrus peel, using the maceration technique. The composition of 80% ethanol was the most efficient at 40 °C in a shaking water bath for 20 h, using a 1:15 sample/solvent ratio. Similar results were obtained for CGAs extraction by maceration starting from artichoke bracts and stems [30], and potato peel [48]. In both cases, the extractions were performed in a heating water bath, and a higher yield of hydroxycinnamic acids was obtained using 80% ethanol as an organic solvent. This trend was analogous to the behavior described by the Soxhlet extraction. In fact, 70% ethanol was more promising both than 50% and 96% ethanol in the recovery of CGAs from sunflower by-products [53] and tomato peel extract [39], respectively. Differently from the above reported works, lower percentages of ethanol (15–20%) were used to recover CGAs from spent coffee grounds at 60 °C for 15 min [42]. The optimal solvent composition of 20.3% ethanol or 100% water was indicated by means of a response surface methodology (RSM) in the maceration of walnut leaves to recover 3-*O*-CQA [35]. These different trends in the effect of organic solvent composition could be explained by the different nature of the treated food matrix, which sometimes could require more water for enhancing the penetration of the solvent in the plant cell matrix. As an example, water was more suitable for CGAs recovery from coffee husk [41] than ethanolic solutions [23] or water acidified with citric acid up to 2%. This addition negatively affected the recovery of the total phenolic acid content of husk, due to the easy degradation of CGAs in acidic conditions [59]. Therefore, for extracts particularly rich in CGAs, as those obtained from coffee by-products, it is very important to consider the acidity of the extraction mixture. 

In recent years, Natural Deep Eutectic Solvents (NaDES) have received great attention as innovative green extraction media, able to replace harsh organic solvents in the extraction of phenolic compounds [60,61]. NaDES are stable eutectic mixtures composed of a hydrogen bond acceptor (HBA) and a hydrogen bond donor (HBD). Recently, Ruesgas-Ramón et al. [62] tested six different DES using heat-stirring assisted extraction (1 h, 60 °C) to extract CGAs from coffee and cocoa by-products. The extraction yield was compared with the results obtained in the same experimental conditions using 70% ethanol. Lactic acid: choline chloride DES (2:1 molar ratio) was the most promising solvent mixture, as an alternative to the hydro-alcoholic mixture, even if it led to a lower extraction yield. 

Working temperature and time are two other key factors highly affecting the recovery of CGAs when conventional hydro-alcholic extractions are performed. Generally, a longer time is required when operating at room temperature, up to 24 h for blueberry leaves hydro-alcholic maceration [31] and 7 days for mango leaves [63]; a shorter time could be enough using the Soxhlet method, as for example in the case of sunflower by-products (7 h). Heating enhances the extraction of bioactives and can reduce extraction times. In fact, temperatures in the range 60–75 °C increased CGA extraction yields, by consequently reducing the extraction time, for potato (120 min) [48], artichoke waste (60 min) [30], walnut leaves (54.3 min) [35], cauliflower and celery waste (20 min) [58], and spent coffee grounds under stirring (15 min) [42]. This could be explained by the fact that employing higher temperatures increases water diffusivity and phenolic compounds solubility, decreases solvent viscosity and surface tension, and helps to weak phenolic-polysaccharides and phenolic–proteins linkages. Thus, these effects favor the migration of bioactives into the extraction solvents [41,51,64]. Hence, the working temperature has a prominent role in CGAs extraction. Recently, Silva et al. [23] detected hydro-alcoholic extracts in coffee husk obtained using a conventional heated assisted extraction at 60 °C a higher chlorogenic acid content than the one obtained with ultrasound-assisted extraction (UAE) at 35 °C. However, temperature values higher than 60–70 °C could lead to intramolecular CGAs isomerization and transesterification or degradation, because of the thermolabile nature of these compounds. In fact, in the range 100–200 °C, in acidic water under reflux for 5 h, increasing the temperature led to an increase in the number of compounds originating from 5-CQA; the new compounds included the isomerization products, i.e., 3-CQA and 4-CQA, and different adducts derived by the reaction with acidic water [65]. Despite the controlled application of heat to favor the extraction of phenolic compounds, conventional approaches are still considered time-consuming processes with large energy expenditure. 

The sample-to-solvent ratio (S/L) is also an important parameter because it is responsible for the creation of a driving force between the solid and the bulk liquid that leads to the extraction from a plant matrix [66]. Usually, it changes in relation to the nature of the food waste. Generally, the extraction yield increases with the use of low S/L because of the molecule concentration and the reduction of saturation effects [64]. This finding was confirmed by Rebollo-Hernanz et al. [41] for the recovery of hydroxycinnamic acids from coffee husk: the yield was higher when 0.02 g dw/mL was used (a more marked enhance of the mass transfer). Another example is represented by the study of Baiano et al. [58], who tested three different S/L ratios, namely1:1, 1:2, 1:4 (*w/w*), for the extraction of phenolic compounds from different vegetable waste matrices. The highest recovery of CGAs was obtained for cauliflower and celery waste using 1:1 (*w/w*) and 1:2 (*w/w*) ratio, respectively. Beyond this ratio, the CGAs extraction yield did not increase, probably because the limit value in the concentration gradient between the solid and the liquid phases was reached, and no more driving force for the extraction process was present. Other authors preferred 1:10 [23], 1:20 [48] or 1:30 g/mL [30], and the optimization of extraction yield was based on the other above-quoted parameters. 

Finally, it was shown that a pretreatment of the matrix, such as milling [41] or drying [23], is also useful to increase the CGAs extraction yield.

## 4. Innovative Green Extraction Techniques

Despite the fact that conventional extraction methods are of practical application and still extensively used to obtain CGAs from food waste, they require a considerable amount of solvent, long extraction times, and frequently expose thermolabile compounds to a higher risk of thermal degradation [5]. In the attempt to overcome these limitations and develop more sustainable approaches, innovative extraction techniques have been proposed: the most promising approaches are discussed in the paragraphs below (Table 2).

### 4.1. Microwave Assisted Extraction (MAE)

**Figure 3:**
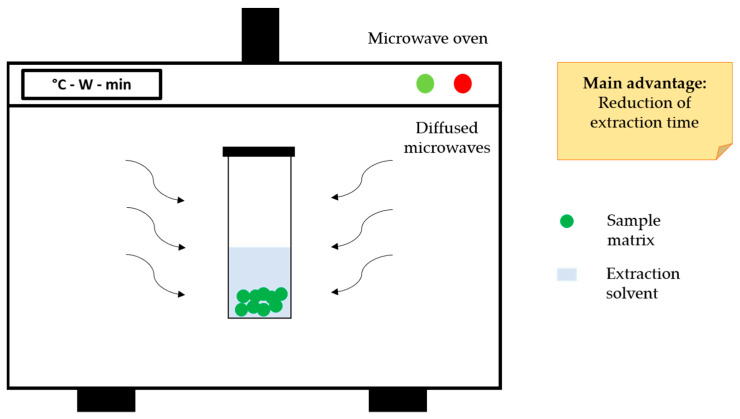
Schematic representation of typical MAE equipment.

Over the last few years, MAE was reported as an innovative green extraction technique for the recovery of CGAs: it is a valid alternative to conventional extraction approaches because it is time-saving, requires a low amount of solvent and gives high extraction yields with good reproducibility. The main disadvantages related to this technique are the poor efficiency towards volatile compounds and high equipment cost. Heating is based on the direct effect of microwaves on molecules by two mechanisms of energy transfer, namely dipole polarization and ionic conduction. Due to their electromagnetic components, microwaves penetrate the plant matrix and interact with polar molecules, leading to an increase of the pressure inside the plant cells [68,69]. This mechanism induces alteration at the cellular level, promoting the breaking of cell walls and the subsequent release of phytochemicals, as demonstrated by scanning electron microscopy analysis performed after microwave irradiation on sunflower by-products, cocoa bean shell, and pomegranate peel [51,53,70] (Figure 3).

Considering the high number of different factors affecting this extraction process, the use of both experimental design and response surface methodology is proved to be a useful tool for such studies [71]. As in conventional extraction procedures, the most important factor affecting MAE is the solvent selection. In this case, polarity is directly linked to solvent dielectric properties, measured through its dielectric constant and dissipation factor, which affect the absorption of microwave energy [31,68,72]. In particular, the homogeneous distribution of heat through the matrix and the consequent increase in the solute extraction yield is facilitated using solvents with high dielectric constant and high dissipation factor [68]. For the recovery of CGAs, water-organic solvent mixtures are reported to be more efficient than pure solvents. Commonly used organic solvents are methanol [70,73] or acetone [74,75,76], but ethanol is preferred for nutraceutical purposes [31,48,68]. Ethanol is considered a stronger energy absorbent compared to water, so the ability of the mixture to absorb energy and convert it into heat is expected to be favored by increasing the ethanol percentage in the mixture [72]. In fact, it is generally reported that the extraction efficiency of CGAs increases by increasing the ethanol percentage in the hydro-alcoholic solvent; however, the efficiency decreases in the presence of very low amounts of water. Wu et al. [47] investigated the effect of the variation of ethanol percentage in the extraction mixture (20%, 40%, 60%, 80%) on the extraction yield of polyphenols and especially of CGAs and caffeic acid from potato downstream wastes. An orthogonal array design was used, and the yield extraction efficiency reached a plateau using 60% ethanol; for higher concentration values, the yield decreased, slowly up to 80% ethanol, and then drastically. This trend was confirmed by a recent study on the extraction of CGAs from sunflower by-products by Náthia-Neves et al. [53]. A water-ethanol 30:70, *v/v* mixture was the most effective, and the CGAs concentration decreased by increasing the ethanol concentration from 70% to 100%. Similarly, the recovery of CGAs from artichoke by-products using pure ethanol was less efficient than using a 50% hydro-alcoholic mixture [45]. Based on the above-mentioned results, the optimal ethanol percentage for CGAs extraction using MAE could be considered in the range 50–70%. 

Mellinas et al. [51] suggested that the pH of the extraction solvent could also highly affect the extraction of CGAs. The effects of different pH values (pH 2, 7, and 12) were monitored in the aqueous extraction of CGAs from cocoa bean shell waste. As shown by SEM analysis, the alkaline condition (pH 12) induced the formation of a crinkled surface characterized by the presence of smooth pores promoting, respectively, the rupture of the cell walls and the release of CGAs. 

Recently, the use of NaDES combined with microwave irradiation was considered as a good alternative to improve the extraction of phenolic compounds from cocoa bean shell. Sixteen different choline chloride (ChCl)-based DES, and 1:1 molar ratio ChcCl: lactic acid (LA,) or 1:2 molar ratio ChCl:urea (U), with the addition of 10% and 50% water, respectively, appeared to be promising solvents to recover CGAs [77]. 

The working temperature is also an important factor for this approach. Usually, high temperature values increase the diffusivity of the solvent into the matrix and the partition of the solutes into the solvent, favoring an increase in the extraction yields. However, the working temperature should be carefully controlled to avoid intramolecular isomerization, transesterification and thermal decomposition or degradation of thermolabile compounds, such as precise CGAs [47,48,65]. In fact, Liazid et al. [78] demonstrated that at 500 W, the highest concentration of caffeic acid, *p*-coumaric acid, ferulic acid, and sinapic acid was obtained by operating at 50 °C for 20 min, while by increasing temperature up to 100 °C, no significant difference was registered. Conversely, heating from 125 to 175 °C, a meaningful reduction in the concentration was noticed. The different behavior of CGAs and other classes of polyphenols could suggest a possible relationship between chemical structure and stability during MAE extraction. A lower substitution extent on the aromatic ring could be correlated to higher stability, and the presence of hydroxyl groups could give a higher susceptibility towards thermal degradation with respect to other substituents. The results obtained by Mena-García et al. [45] confirmed this trend: by increasing the temperature from 50 to 69 °C, the extraction extent of CGAs from artichoke waste increased, but a further increase in temperature had a negative effect, and 120 °C was outlined as the best temperature for extracting the total phenolic content. Similar results were reported for the recovery of CGAs from tomato waste (55 °C for 5 min for CGAs, but 90 °C for total phenolic content) [37] and walnut leaves (61 °C for CGAs and 107 °C for the overall phenolic compounds) [35]. Guglielmetti et al. [24] investigated the CGAs extraction yield between 37 and 80 °C, registering a better CGAs extraction efficiency at 37 and 55 °C followed by a decline at 80 °C. Considering that the use of higher temperature can promote the extraction of other compounds than CGAs [79], it may be concluded that optimum extraction conditions are not homogeneous for all the polyphenolic classes, and a higher CGA extraction yield can be obtained in the range 50–70 °C. 

The irradiation power is closely connected to temperature. In fact, it can enhance the interaction extent between the electromagnetic field and the material, facilitating the penetration of the solvent into the plant matrix and hence induce heating. Because of that, increasing the microwave power to higher levels means to increase the temperature inside the microwave [80]. This is the reason why different authors only took into consideration one of the two parameters of temperature and irradiation power. Náthia-Neves et al. [53] investigated the impact of different extraction powers (100, 200 and 300 W) on the CGAs recovery from sunflower by-products. The optimum CGAs extraction yield was achieved at 200 W, probably because the microwave power promoted the rupture of the cell walls; conversely, at 300 W, when the temperature rose up to 105 °C, thermal degradation reduced the extraction capacity, thus confirming the previous findings of Liazid et al. [78]. These results highlighted the importance of finding an optimal combination of microwave power and temperature for the recovery of a high amount of CGAs. 

Extraction time is another factor interacting with microwave power and it can be optimized to avoid thermal degradation. In fact, exposure to microwave irradiation for longer times generally reduced the extraction yield of phenolic compounds [81]. In particular, the recovery of CGAs from blueberry leaves was very similar, applying 71W for 24 min or 142.1 W for 4 min [31], but irradiation power of 300 W even only for 120 s may lead to overheating with decomposition of the phenolic compounds [53]. However, using 400–900 W for shorter times allows setting an optimal temperature, avoiding overheating and thus optimizing CGAs extraction [35,37,45,51,80]. Several studies reported that the extraction of CGAs from food waste reached a saturation point in a very short time, generally in the range 0.5–5 min [31], as confirmed for tomato peel (5 min) [37], artichoke waste (3 min) (45), potato peels and olive leaves (2 min) [47,82], and sunflowers by-products (30 s) [53]. These results outlined the ability of microwave-based extraction to recover bioactives in shorter times than conventional procedures [31,40,47,48,51]. Therefore, this technique can be considered as an innovative sustainable extraction method useful for producing high-quality extracts while reducing processing times. Furthermore, the use of shorter times allows reaching higher temperature values without thermal degradation, making MAE extraction a technique suitable for thermolabile compounds, such as CGAs. 

Finally, the last parameter affecting MAE extraction is the solvent-to-solid ratio, which is strictly correlated to the homogeneous distribution and exposure of the solute to microwaves and the creation of a mass transfer gradient responsible for driving bioactives outside the food matrix [68]. The lowest solvent-to-sample ratio (<1:5, *v/w*) is related to a decrease in the solvent surface area and to a lower penetration of microwave irradiation into the suspension [53]. On the contrary, the highest solvent-to-solid ratio (>1:50, *v/w*) could also reduce the recovery due to a longer time needed to reach the selected temperature [47]. The effect of the solvent-to-raw-material ratio depends on the nature of the tested food waste. Based on the studies collected here, the higher CGAs extraction yield for sunflower by-products, potato waste, and cocoa bean shell waste were obtained using 1:10, 1:40, and 1:25 *v/w*, respectively [47,51,53].

### 4.2. Ultrasound-Assisted Extraction (UAE)

UAE is considered a green and sustainable technique, alternative to conventional extraction methods, because it is a time saving process that allows to reduce or eliminate toxic solvents consume and recover good amounts of the compounds of interest [33]. Other advantages consist in simple equipment, versatility, lower energy output, high extraction efficiency in a short time, and no thermal effect [43,83]. This last characteristic makes this method suitable for the extraction of polyphenols, including CGAs. The main disadvantage related to this technique is the scale up, and therefore, its diffusion at the industrial level is very low [54]. 

**Figure 4:**
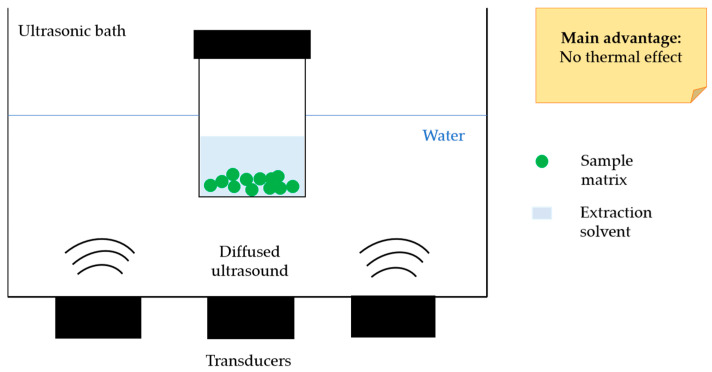
Schematic representation of typical UAE equipment.

Ultrasounds are mechanical waves characterized by different physical proprieties, such as frequency, power, intensity, and amplitude [84], which deeply influence the ex-traction performance and should be investigated to optimize the extraction process (Figure 4).

The propagation of ultrasound waves in a liquid causes the acoustic cavitation phenomena consisting of formation, growth, and explosion of cavitation bubbles [84]. More specifically, ultrasound produces a displacement of the molecules present in the liquid media around their main position because of the alternating of compression and rarefaction phases of waves. When the negative pressure exerted by ultrasound is higher than the cohesive forces among molecules, the formation of a cavity in the liquid (the so-called cavitation bubble) occurs [85]. The cavitation bubbles can be stable or transient. The stable ones endure many rarefaction/compression cycles because they can elastically oscillate around an equilibrium size while the transient bubbles last for few cycles during which they increase their size until they violently collapse [84]. The explosion causes the generation of local extreme temperature and pressure conditions and, when this phenomenon occurs in the presence of a plant matrix, a high-speed jet of solvent against the surface of plant cells is generated; this impact can damage the cell wall, facilitating the release of bioactive compounds [86,87]. UAE extraction efficiency is thus due to the mechanical effects of ultrasound (described above), which allows a deep penetration of the solvent into the plant material and promotes a continuous circulation of new solvent, consequently increasing the mass transfer from solid to liquid phase [79,87]. Therefore, UAE could be an interesting technique to obtain extracts rich in CGAs using lower temperatures than the conventional extraction approaches [88]. 

Ultrasound can be directly or indirectly applied to the sample. The ultrasonic bath is the most common ultrasonic device consisting of a water bath equipped with one or more transducers as a source of ultrasounds. In this case, ultrasounds are delivered through water to the wall of the sample device, up to the sample molecules; conversely, the ultrasonic probe is composed of a transducer directly immersed in the sample, and it reduces the ultrasonic energy loss, improving the reproducibility of the performance compared to the ultrasonic bath, which, on the other hand, allows the operator to handle many samples simultaneously and is very cheap [85].

Ultrasound frequency is basically kept constant at a fixed value between 20 and 100 KHz, which is the range necessary to achieve cavitation. Frequency is inversely correlated to the rarefaction phase duration. In fact, at high frequency, the succession of rarefaction/compression phases becomes so short that the cavitation bubbles cannot be generated and cavitation is no longer obtained [85]. The ultrasound power output is the effective energy transferred to the sample and is a key parameter to be optimized in order to use only the minimum energy required to obtain the best results [84]. The extraction yield of CGAs from spent coffee grounds increased by working in the range 100–250 W using a probe-type sonicator, after which the response decreased [43]. Similar results were obtained by Rabelo et al. [46], who investigated the effect of different power values, i.e., 0, 240, 480, and 720 W, in the extraction of CGAs from artichoke bracts; in fact, also in this case, starting from 240 W, a further enrichment of the extract in CGAs was no longer possible. A power of 122.44 W was the best value to maximize the CGAs extraction yield from almond skin waste, too [32].

Other researchers investigated the mechanical effect of ultrasounds, studying the ultrasound intensity or the amplitude. The ultrasound intensity, expressed as the ultrasound power transmitted per cm^2^ of the emitting surface of the transducer (W/cm^2^), is directly correlated to the amplitude of transducer vibration [84]. Zardo et al. [79] studied a UAE method for extracting CGAs from sunflower seed cake, which is the main by-product obtained in the sunflower oil production. HPLC analysis indicated 5-CQA as the main CGA present in the extract. Different amplitude values (0, 23, 40, 64, 80 μm, which corresponded to 0, 11, 14, 30, 43 W/cm^2^, respectively) were investigated. Results indicated that ultrasonic amplitude (UA) had a positive and linear effect because extraction yield increased by increasing UA, even if UA had a less significant influence on extraction yield (*p* value > 0.05) than ethanol concentration and temperature, probably because vegetable cells are already damaged by the oil extraction process, and therefore, the mechanical effect caused by ultrasound are less important. Interestingly, Alves Filho et al. [83] performed a CGAs water extraction from potato peel focusing on the ultrasonic power density, namely the power transmitted per liter of solvent. Three different conditions were applied, i.e., 20, 35, and 50 W/L, and the milder conditions enhanced the extraction of 3,4-diCQA and 3,5-diCQA, but not of tri-CQAs, which partially hydrolyzed to 3,4-CQA. The exposure to 50 W/L power density allowed the extraction of higher amounts of 3-CQA and 3-caffeoyl-4-feruloylquinic acid, a substance not extracted without sonication and applying the lowest power density, but unfortunately, 3,4-diCQA hydrolyzed, giving rise to 3-CQA. These results highlighted the importance of setting up appropriate values for power density and pH, depending on the waste matrix and its composition in CGAs.

In addition to the physical effects of waves, temperature, time, kind of solvent, and solvent-to-solid ratio are important parameters to be controlled to optimize CGAs extraction from waste also in UAE.

The solvent highly affects the extraction efficiency: polarity, viscosity, vapor pressure, and surface tension are crucial factors in the solvent selection [23]. In fact, if polarity is related to the composition of extract, viscosity and surface tension can hinder the propagation of ultrasounds and the cavitation phenomena, while high vapor pressure reduces the mechanical effect of ultrasounds [84]. The selection of the proper water/solvent ratio in the hydro-alcoholic mixtures is important because water has a swelling effect on the matrix, increasing the contact surface area between the plant matrix and solvent [33], while ethanol due to its lower dielectric constant can modulate the polarity of the aqueous solution, thus enhancing CGAs diffusion and extraction [79]. Moreover, the hydro-alcoholic solutions have lower viscosity than the aqueous mixtures, resulting in a better mass transfer of compounds. Zardo et al. [79] identified 43% ethanol as the optimal composition of the solvent system for extracting CGAs from sunflower seed cake. A similar solvent composition was also used for different types of tobacco wastes (40–55% ethanol) [54]. Conversely, a higher ethanol percentage was needed to recover CGAs from carrot pomace [33] and artichoke bracts (80% and 75%, respectively) [46]. In recent years, there has been a growing interest in developing organic solvent-free extraction methods and in testing alternative less toxic and more environmentally friendly solvents systems. Alves Filho et al. [83] tested acidified water for the CGAs extraction from potato peel obtaining good results. Punzi et al. [28] optimized a UAE method using 100% pure water for artichoke waste. Other alternative solvents were aqueous PEG solutions: in particular, a 40% aqueous PEG400 solution allowed a higher CGAs recovery efficiency from almond skin than ethanol, methanol, acetone and PEG with different molecular weights [32]. An aqueous-glycerol mixture in which a smaller amount of organic solvent was required to reduce water polarity (the dielectric constant is lower for glycerol than for water) was also tested as an alternative to an aqueous-ethanol one, giving similar CGA extraction yields from potato waste when operating with an ultrasonic bath at 37 kHz, 140 W, 35 W/L [67]. 

Beta-cyclodextrin (β-CD) water solutions were also a good alternative solvent to recover phenolic compounds [34]. β-CD are cyclic oligosaccharides recognized as GRAS, widely used in the food industry to enhance stability, solubility, and bioavailability of food ingredients [89]. β-CD structure is constituted by a hydrophilic surface and a hydrophobic internal cavity, which can host non-polar molecules with molecular weight ranging from 200 to 800 g/mol and improve their solubility in water. The molecular weight of phenolic acids, including CGAs, is about 354–190 g/mol, and therefore, β-CD is suitable to form an inclusion complex. The results obtained [34] using 1.8% β-CD solution at 55.76 °C for 15.38 min in an ultrasonic bath at 100 W and 40 kHz indicated that β-CD solution can be efficiently used as a solvent system to recover CGAs from pomegranate peel.

DES were used by Fanali et al. [21]. The solvent mixture consisting of betaine as acceptor and triethylene glycol as donor of hydrogen bonds, ratio 1:2, extracted a higher CGA amount from spent coffee ground than a conventional water–methanol mixture (70:30 *v/v*), by using the same experimental conditions (200 W, 37 kHz). A very similar approach was also used by Yoo et al. [22] but selecting a different DES, i.e., 1,6 hexanediol-Choline Chloride, 7:1 molar ratio. A key point in the use of DES is represented by the optimization of water content to be added in order to reduce the viscosity of the extracting mixture [90], which is generally selected by applying an experimental design, as in the case of Fanali et al. [21] (30% water, *v/v*) and Yoo et al. [22] (32.5% water, *v/v*).

Considering the temperature, as previously reported, the passage of ultrasound does not cause heating of the liquid media, and therefore, the low working temperature could be considered a great advantage, especially in the extraction of thermally sensitive compounds such as CGAs [83]. Anyway, many researchers analyzed the effect of this parameter to perform the extraction process with ultrasounds. For example, Jabbar et al. [33] studied the optimal extraction condition of CGAs from carrot pomace, a by-product of carrot juice production, using an ultrasound probe (750 W, 20 kHz, 70% amplitude level, and 40 W/cm^2^ ultrasound intensity). Temperature and ethanol percentage had a significant positive effect on the extraction yield, and 20 °C was selected as the best operative condition in the range 10–60 °C, thus confirming that UAE are effective in extracting CGAs even at low temperatures. This conclusion was supported by the CGAs recovery study from potato peel waste, which detected room temperature (25 °C) as the best condition [83]. A higher temperature (45 °C) was optimal to extract CGAs from spent coffee ground and from sunflower seed cake (70 °C). In the first case, the correlation between cavitation and solvent vapor pressure was interestingly highlighted: an increase in the extraction temperature caused a corresponding increase in the solvent vapor pressure, resulting in a lower amount of energy released when bubbles collapsed and consequently reducing the mechanical effect of ultrasounds [43]. In the second case, the thermal effect prevailed over the mechanical one because the sample matrix is already damaged; therefore, higher temperature promoted the extraction by reducing solvent viscosity, enhancing CGAs solubility and diffusion rate [79]. A temperature lower than 60 °C is recommended even if the type of waste highly affected the setup of temperature, as demonstrated by Banožić et al. [54] for tobacco wastes (midrib, dust and scrap) applying 37 kHz, 50 W, and working in the 30–55 °C temperature range. Temperature becomes extremely relevant when dealing with a deep eutectic solvent: in fact, the main problem of this solvent is the high viscosity, which can be reduced by adding water and using a high working temperature [21,22,90]. 

A prolonged sonication could lead to the degradation of bioactive compounds, and therefore, time is a critical factor in UAE [83]. However, the use of ultrasound allowed to reduce the CGAs extraction times in comparison with conventional methods [28,33,43,44,46,54] because it is easier to extract bioactive compounds when the sample cells are already broken [79], thus avoiding degradation. 

UAE is an efficient extraction method even at a low solvent-to-solid ratio and this feature makes UAE an eligible procedure to reduce solvent consumption and develop green processes. This ratio resulted in significant extraction from almond skin (with a weak negative effect) and potato peel, and the highest recoveries were obtained using 1:20 and 1:84 g/mL, respectively [32,67]. Conversely, sometimes the solvent-to-solid ratio was not a significant variable in CGAs recovery, as demonstrated by Al-Dhabi et al. [43] for spent coffee ground, even if it had a mild positive effect on the extraction yield, which was optimized using the 1:17.5 g/mL ratio. Several other studies did not examine the effect of this variable, but a fixed ratio was used [21,46,50,79]. 

### 4.3. Supercritical Fluid Extraction (SFE)

**Figure 5:**
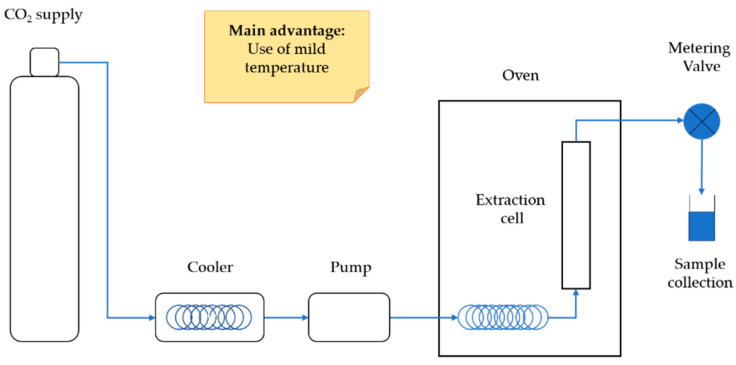
Schematic representation of typical SFE equipment.

SFE is based on the use of supercritical fluids (SF) as extracting solvents. Due to their liquid-like density, low viscosity, and high diffusivity, typical of a gas phase, SF can easily diffuse through solid materials, enhancing a mass transfer rate and solute diffusion [91,92,93]. SF density can be modified by changing pressure and temperature conditions, thus affecting fluid solvating power and analyte solubility [94]. Specifically, when increasing density, the pressure increases, resulting in a better SF solvation power, while high temperature reduces solvent density but simultaneously enhances solute diffusivity [95]. SFE is considered a sustainable method because it involves the use of solvents recognized as safe and is characterized by low extraction times. SFE technology is widely used at the industrial scale, for example, in the decaffeination of coffee and tea, and in the extraction of essential oils and fish oils or flavors from natural sources and spices [96].The most commonly used SF is carbon dioxide, which accounts for many advantages: it is safe for human health, non-polluting, and easy to be removed by decompression because it returns to the gaseous state, reducing the number of steps needed for the sample post-extraction purification process. Moreover, its low critical temperature (31 °C) allows the extraction of thermally labile compounds such as CGAs, also preventing sample oxidation because the process is carried out without air contact [92,94,97]. However, carbon dioxide is not very efficient at extracting polar compounds like CGAs, and therefore, an organic modifier or cosolvents such as ethanol or methanol are required to increase solvent polarity [94]. The optimal amount of organic modifier should be studied and optimized to maximize the recovery of the compounds of interest. Therefore, despite several advantages and its potentiality, in recent years, SFE has not been widely used for CGAs extraction from agri-food wastes and only a few examples of successful application of SFC-CO_2_ were found the in literature, as reported below (Figure 5). 

The effect of SFE-CO_2_ pressure value on CGAs and other polyphenols extraction from tomato skin by-products was investigated by Pellicanò et al. [38]. The extraction was performed at 60 °C, using 2 mL/min flow rate for 20–80 min. Pressure levels of 350, 450, 550 bar were tested and the obtained results pointed out that this parameter did not produce a significant variation in CGAs content, differently from what happened for the other polyphenols. Conversely, when olive tree pruning waste, leaves, and exhaust pomace were extracted at 50 °C in the 200-300 bar pressure range for 60 min with the addition of 60% ethanol as cosolvent (1:3 ratio *w/v*), good results in CGAs recovery were obtained, especially for olive tree pruning waste working at 300 bar [56]. Similar experimental conditions were also applied to treat spent coffee grounds and husks, and the best extraction yields were obtained at 60 °C, with working pressures of 300 and 200 bar for husks and spent grounds, respectively [95]. 

Time of extraction, flow rate, and cosolvent type and volume are other important variables to be considered in the setup of the SFE method to extract CGAs [94,97,98].

### 4.4. Pressurized Liquid Extraction (PLE)

**Figure 6:**
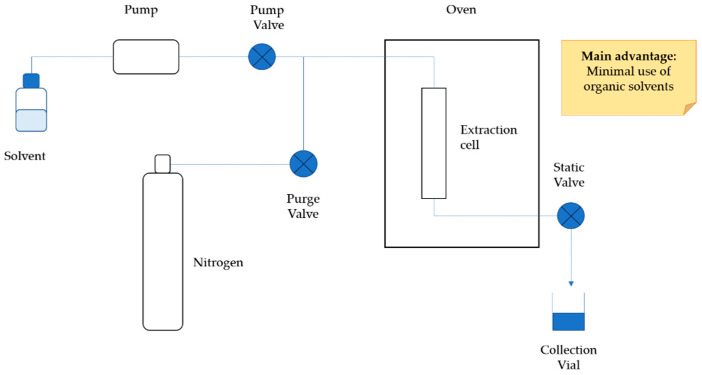
Schematic representation of typical PLE equipment.

In recent years, a novel technique, namely pressurized liquid extraction or pressurized hot water extraction (PHWE), or subcritical water extraction (SWE) [17] emerged. It involves the use of a liquid solvent in conditions of high temperature and high pressure to efficiently extract bioactive compounds [99]. Compared to conventional solid-liquid extraction methods, PLE allows faster extraction with lower consumption of solvent, minimizing the use of organic solvents (it could be solvent free), and has lower environmental impact [57]. PLE uses high pressure to prevent solvents boiling at the extraction temperature [17,100,101] and high temperature to improve bioactives solubility and accelerate their diffusion, reducing viscosity and surface tension [29,57]. One of the main advantages could be considered the use of water as an extraction solvent although the use of 100% water as a solvent is not usually very effective because of its high polarity. Temperature and pressure levels must be maintained under the critical point of water, 374 °C and 22.1 MPa, to keep water in a liquid state during the extraction procedure [102]. A relative water dielectric constant depends on the applied temperature: at room temperature is about 80 and it decreases by increasing the temperature reaching values similar to that of organic solvent, thus facilitating the extraction of fewer polar compounds [103,104]. PLE can be performed in dynamic or in static mode. The dynamic mode consists of a continuous solvent flowing in the extraction vessel, while in the static mode, the solvent volume in contact with the sample for a prefixed time is constant [105,106]. The main advantage of the dynamic mode is that analytes are exposed to high temperatures for a shorter time compared to the static one, with a lower degradation risk for thermally sensitive compounds. Additionally, in the static mode, the analytes diffusion through the solvent can reach an equilibrium, reducing extraction efficiency; to avoid this phenomenon, extraction is carried out for several cycles, adding fresh solvent every time. Conversely, the main disadvantage of the dynamic mode is the high cost [105] (Figure 6).

In CGAs extraction, pressure has only a slight effect on the extraction yield, and therefore, it is usually kept constant, around 60–100 bar [29,49,57,107]. Conversely, temperature and time are critical factors to be considered because high temperature or long exposure time could lead to the degradation of thermally sensitive compounds such as CGAs. 

Apple by-products were extracted by Plaza et al. [107] and by da Silva et al. [57], who investigated the effect of temperature and extraction time (25, 50, 112, 175, 200 °C for 3,5,10,15,17 min) or only temperature (60, 70, 80 °C), respectively. The highest 5-CQA recovery was obtained at 175 °C and for a time duration of 5 min by Plaza et al. [107], and at 60 °C–60 min for apple pomace by da Silva et al. [57] (over 80 °C the yield drastically reduced because of degradation). The different optimal working temperature setup by the two research groups on the same by-products may be attributed to the different applied modes: static exposure for few minutes vs. dynamic mode (2 mL/min) for one hour, respectively. The dynamic approach was also used for developing an SWE method for CGAs recovery from potato peel. In this case, the best conditions to maximize CGAs yield were 160 °C for 120 min, at a 2 mL/min constant flow [49]. Operating at higher temperature induced CGAs degradation. A surface response design was used to study and optimize a PHWE static process to be applied to artichoke by-products. Temperature (60–110 °C), static extraction time (4–10 min), ethanol percentage (0–10% *v/v*), and number of cycles (2–4) were considered as variables. The extraction of 5-CQA and 1,5diCQA were strongly affected by temperature and by the modifier percentage, both having a positive effect. Increasing the temperature, a 1,5diCQA isomerization to 1,3diCQA occurred. Therefore, the optimized extraction conditions, which maximize the recovery of compounds of interest and minimize the extraction of 1,3diCQA were 93 °C, 5 min, 2 cycles, and 10% ethanol *v/v*. The addition of an organic modifier was useful to reduce the extraction temperature since it can modulate the polarity of water and facilitate the solubility in water of bioactive compounds [29].

## 5. Comparison among the Reviewed Extraction Techniques: Is There a Better One to Recover CGAs?

Considering the literature data, it is possible to conclude that there is no approach that can be considered better than the others, and a direct comparison among the extraction techniques is often difficult because their efficiency is closely connected to the characteristics of the tested food matrices and the optimization of the extraction parameters, independent of the type of used method. Instead, a practical comparison among the approaches in terms of the desirable advantages could be useful to guide researchers in choosing the extraction technique (Table 3).

In general, considering the extraction of CGAs in optimal conditions, the use of in-novative approaches could allow for a more efficient recovery. A common trend that can be observed is that when a parameter enhances cell disruption, the total sum of CGAs is higher. MAE, UAE, and PLE are usually more effective compared to conventional ap-proaches because of the direct destruction of the matrix cellular structure induced, respec-tively, by microwaves, ultrasound, and pressure, which lead to a greater release of bioactives into the solvent [31,50,51,53,82,88]. It is interesting in this regard to consider the study by Routeay et al. [31], where this phenomenon is a determinant for the extraction efficiency. The authors compared the MAE, UAE and CSE approaches to recover CGAs from blueberry leaves and found MAE as the most efficient one. Cellular destruction takes place both in MAE and in UAE, but in the present case, the low-frequency level of ultrasound compared to microwave can explain the lower yield of CGAs obtained with UAE. Moreover, Dobrinčić et al. [82] found that MAE, UAE, and PLE are more efficient than CSE for the recovery of total phenolic compounds from olive leaves, confirming the previous studies. However, considering the extraction of CGAs, UAE results to be the most promising approach (0.47 ± 0.02 mg/g dw), followed by MAE (0.44 ± 0.00 mg/g dw), CSE (0.41 ± 0.01 mg/g dw), and PLE (0.39 ± 0.02 mg/g dw). SFE is not widely used to recover CGAs and more data are needed for making exhaustive considerations. Comparing the study by Pellicano et al. [38] with the work by Ničević Grassino et al. [39], the use of SFE extraction allows a higher yield of CGAs from tomato peel with pectin (4.05 mg/kg dw) rather than a conventional extraction method (3.044 mg/kg dw), showing the potentiality of this novel approach. 

However, it is important to underline that the use of an innovative technique is not directly correlated to a higher extraction efficiency. It is crucial to consider the characteris-tics of the food matrix and how it behaves in relation to the extraction parameter changes. For example, the recovery of CGAs from coffee waste matrices seems to be highly influ-enced by temperature, independently from the type of extraction. Guglielmetti et al. [24] found UAE and CSE more efficient than MAE for the recovery of CQAs from coffee silverskin. UAE and CSE are performed at 80 °C and allowed to obtain high yields (2.257 g/kg dw and 2.61 g/kg dw), whereas the optimal conditions for MAE consist of low tem-perature and low power (46 °C and 280 W), resulting in lower yields (1.98 g/kg dw). Sim-ilar results are obtained on coffee husk by Silva et al. [23]. They detected a higher CGA content in coffee husk hydro-alcoholic extracts obtained using a CSE at 60 °C (337.07 ± 9.88 μg/g dw) than those obtained with UAE at 35 °C (304.36 ± 13.03 μg/g dw), even if the mechanical effect of ultrasounds on the vegetable matrix should increase the release of bioactives.

## 6. Conclusions

Nowadays, sustainability is a central issue in several fields, including food and nutraceuticals. Many researchers focus their attention on food waste valorization as a potential source of bioactives whose use could improve human health.

Different types of waste coming mainly from the consumption of artichoke, potato, tomato, and coffee resulted in being promising sources of CGAs, widely known for their beneficial properties. To comply with the principles of a green economy, finding alternative eco-friendly extraction methods to recover CGAs has become a requirement. New innovative green and sustainable techniques emerged, such as MAE, UAE, SFE and PLE, presenting some advantages with respect to the conventional approaches, namely time saving, reduction of the solvent consumption, and high efficiency. This review summarizes the critical parameters affecting CGA extraction, depending on the waste type and the extraction method. In general, solvent selection and temperature control are important factors, independent of the type of extraction. CGAs are polar compounds that require polar solvents to be extracted. A hydro-alcoholic mixture, aqueous solution of glycerol, PEG or β-cyclodextrins and DES emerged as efficient solvents, with the further advantage of being GRAS. Temperature is a key factor in extraction because of the thermolabile nature of these bioactive compounds. Despite the fact that MAE is an approach that implies heating due to the direct effect of microwaves on samples, the use of low power values or short times has been proved not to induce thermal degradation. UAE does not show a thermal effect but exploits the mechanical effect of ultrasounds to efficiently recover CGAs, resulting in a valid alternative to MAE. Moreover, SFE and PLE appear to be promising processes because they allow an efficient extraction of CGAs with minimal use of organic solvents. 

Further investigations are required to promote the scale up and diffusion of these methods at the industrial level as well as to describe and optimize specific controls on extract purity. In fact, the use of herbicides and insecticides is widely diffuse in agriculture, and traces could remain in the final product, compromising its safety.

## Figures and Tables

**Table 1:** Agri-food waste sources of chlorogenic acids (CGAs) and their relative content (referred to as fresh—fw or dry weight—dw).

Source	Type of Waste	CGAs Content	Ref.
Cauliflower	external leaves, stems	21–98 mg/kg fw	[40]
Celery	external leaves, stems	13–19 mg/kg fw	[40]
Coffee	husk	337 μg/g dw ^1^	[23]
		90,567 μg/g dw	[41]
	spent ground	22.08 mg/g dw	[42]
		1.36 mg/g dw	[43]
		1700–1757 mg/kg dw	[20]
		19.6 mg/g dw	[22]
		0.93 mg/g fw	[44]
	silverskin	1.06–2.68 g/kg dw	[24]
Artichoke	bracts	3.73 mg/g dw	[30]
		12.98 mg/g dw	[45]
		0.02–16.47 mg/g dw	[46]
	leaves, stems	3–16 mg/g dw	[29]
	leaves, outer bracts, stems	74.2 mg/kg dw	[28]
	stems	8.86 mg/g dw	[30]
	stalks	1.56 mg/g dw	[45]
	receptacles	32.10 mg/g dw	[45]
	leaves	2.39 mg/g dw	[45]
Tomato	peel	6–62 mg/kg fw	[36]
		3.04–4.54 mg/g dw	[38]
		4.05 mg/kg dw	[37]
Potato	peel	6.63 mg/g dw	[47]
		0.032–1.03 mg/g dw	[48]
		0.15 mg/ g dw	[49]
		1.3–4.1 mg/g dw	[26]
Walnut	leaves	8.6–10.7 mg/g dw	[35]
Blueberry	leaves	47.271–51.631mg/g dw	[31]
Mango	peel	33.97 μg/g dw	[50]
Carrot	pomace	17.79 mg/g dw	[33]
Cocoa	bean shell	0.76 mg/g dw	[51]
Citrus	peel	20.52 μg/g dw	[52]
Sunflower	by-products	3.2–15 mg/g dw	[53]
Almond	skin	15.99 mg/g dw	[32]
Tobacco	scrap, midrib, dust	36.4–804.2 μg/g dw	[54]
	residual stalks	1198.0–1998.6 μg/g dw	[55]
Olive	exhaust olive pomace	0.31 mg/g dw	[56]
	tree biomass	0.24 mg/g dw	[56]
	leaves	0.09 mg/g dw	[56]
Apple	pomace	0.718 mg/g dw	[57]
Pomegranate	peel	3.07 mg/g dw	[34]

^1^ content expressed as 3-CQA.

**Table 2:** Overview of the most relevant green extraction methods and related optimal extraction conditions for CGAs recovery from agri-food waste.

Source	Type of Waste	Extraction Method	Optimal Extraction Conditions	Ref.
Coffee	Husk	UAE	50% EtOH, 35 °C, 1 h, 1:10 *w/v*, 40 kHz, 220V	[23]
		CSE	50% EtOH, 60 °C, 1 h, 1:10 *w/v*	[23]
	Spent ground	CSE	15–20% EtOH, 40–60 °C, 15–25 min, 0.30:25 *w/v*	[42]
		UAE	100% EtOH, 40 °C, 34 min, 1:17 *w/v*, 20 KHz, 244 W	[43]
		UAE	betaine:triethylene glycol 1:2, 30% water, 65 °C, 20 min, 1:15 *w/v*, 37 KHz, 200 W	[21]
	Silverskin	MAE	60% EtOH, 43.5 °C, 31.5 min, 1:35 *w/v*, 280 W	[24]
		CSE	60% EtOH, 67.5 °C, 36.5 min, 1:35 *w/v*	[24]
Artichoke	Bracts, stems	CSE	80% EtOH, 60 ± 0.1 °C, 60 min, 0.5:15 *w/v*	[30]
	Leaves, external bracts, stalks, receptacles	MAE	50% EtOH, 50–69 °C, 3 min, 0.3:10 *w/v*, 900 W	[45]
	Internal and external bracts	UAE	75% EtOH, 25 °C, 10 min, 1:10 *w/v*, 20 kHz, 240 W	[46]
	Outer bracts, stems	UAE	100% water, 60 min, 1:3 *w/v*, 25 kHz, 1200 W, 50 W/L	[28]
	Bracts, leaves	PHWE	10% EtOH, 93 °C, static time 5 min, 2 cycles, 103 bar	[29]
Tomato	Peel	MAE	70% EtOH, 55 °C, 5 min, 1: 50 *w/v*, 0–500 W	[37]
Potato	Peel	MAE	60% EtOH, 80 °C, 2 min, 1:40 *w/v*, 300 W, 120 rpm magnetic stirring	[47]
		UAE	83% glycerol, 80 °C, 90 min, 1:81 *w/v*, 37 kHz, 140 W, 35 W/L	[67]
		SCW	100% water, 160 °C, 60 min, 60 bar, 2 mL/min	[49]
Walnut	Leaves	MAE	100% water, 61.1 °C, 3 min, 0.030:1 *w/v*, 850 W, 600 rpm magnetic stirring	[35]
		CSE	100% water, 29.9 °C, 150 min, 0.030:1 *w/v*, 600 rpm magnetic stirring	[35]
Blueberry	Leaves	MAE	30% EtOH+ 0.03% citric acid (1.5 M), 4 min, 0.5:80 *w/v*, 141.1 W	[31]
Cocoa	Bean shell	MAE	100% water, pH 12, 97 °C, 5 min, 0.04:1 *w/v*, 500 W, 400 rpm magnetic stirring	[51]
Sunflower	By-products	MAE	70% EtOH, 200 W, 30 s, 1:10 *w/v*	[53]
Citrus	Peel	CSE	80% EtOH, 40 °C, 20 h, 1:15 *w/v*	[52]
		UAE	80% EtOH, 45 °C, 60 min, 1:20 *w/v*, 35 kHz	[52]
Cauliflower	External leaves, stems	CSE	100% water, 70 °C, 20 min, 1:1 *w/w*	[58]
		MAE	100% water, 4 min, 1:1 *w/w*, 750 W	[58]
Celery	External leaves, stems	CSE	100% water, 70 °C, 20 min, 1:2 *w/w*	[58]
		MAE	100% water, 4 min, 1:2 *w/w*, 750 W	[58]
Tobacco	Midrib, dust, scrap	UAE	40–55.43% EtOH, 30.14–53.59 °C, 15.19–30.31 min 1:10–11 *w/v*, 37 kHz, 50 W	[54]
Carrot	Pomace	UAE	80% EtOH, 20 °C, 10 min, 1:50 *w/v*, 20 kHz, 70% amplitude level, 48 W/cm^2^	[33]
Almond	Skin	UAE	50% PEG200, 75 °C, 20 min, 1:20 *w/v*, 40 kHz, 120W	[32]
Apple	Pomace	PLE	100% water, 60 °C, 60 min, 100 bar, 2 mL/min	[57]
Olive	Tree biomass	SFE	CO_2_+ 60% EtOH, 50°C, 60 min, 1:3 *w/v*, 300 bar	[56]

**Table 3:** Practical comparison of the desirable advantages among the considered extraction approaches.

Desirable Advantages	CSE	MAE	UAE	SFE	PLE
Low cost	+	−	+	−	−
Easy to perform	+	+	+	-	−
No thermal effect	−	−	+	+	−
Time-saving	−	+	+	−	+
Low solvent consumption	−	+	+	+	+
Low energy consumption	−	+	+	-	−

## Data Availability

Not applicable.
